# Comparison of “Lumipulse anti‐*Treponema pallidum*” and “Architect Syphilis TP” and further examination

**DOI:** 10.1002/jcla.23194

**Published:** 2020-01-24

**Authors:** Jianbo Liang, Jianxin Wan, Chumei Huang, Gaorong Cai, Laisheng Li, Min Liu

**Affiliations:** ^1^ Department of Laboratory Medicine The First Affiliated Hospital of Sun Yat‐sen University Guangzhou China

**Keywords:** anti‐*Treponema pallidum*, syphilis, *Treponema pallidum*

## Abstract

**Background:**

Syphilis is a sexually transmitted disease caused by *Treponema pallidum* (TP) infection. In recent years, diagnostic reagents with fully automated immunoassay instruments have become mainstream. However, these positive screening tests with high sensitivity, which are performed with full automation, need confirmation by *Treponema pallidum* particle agglutination (TPPA) to be judged as positive.

**Methods:**

We evaluated the diagnostic performance of Lumipulse G TP‐N assay (Lumi‐TP, Fujirebio Inc) in 223 preselected TP‐positive samples and 1041 TP‐negative samples, compared it with that of the TP gold standard test (TPPA) and Architect Syphilis TP test (Archi‐TP, Abbott).

**Results:**

The concordance rates for the results for the positive and negative samples between Lumi‐TP and TPPA were 100%. On the other hand, the rates for the results between Archi‐TP and TPPA were 100% for positive samples and 99.14% (1032/1041) for negative samples. Correlation tendency and rate between Archi‐TP and Lumi‐TP were 2.549 and 0.841 in positive specimens up to 160 detected values in Lumi‐TP. However, the detection value of Archi‐TP reached a plateau when it exceeded about 40. Furthermore, according to the comparison of each value obtained from Archi‐TP and Lumi‐TP with the strength of the staining of each line in the immune‐chromatography assay kit, ESPLINE TP (Fujirebio Inc) for TP major antigens, Tp15‐17 and TpN47, it was found that Lumi‐TP obtained higher values than Archi‐TP, particularly for TpN 47.

**Conclusions:**

Lumi‐TP has high specificity and is useful not only for screening but also for determining the amount of anti‐TP antibodies.

## 1 INTRODUCTION

Recently, the epidemiological re‐emergence of syphilis has occurred in many countries, including China.^1^ If the diagnosis and subsequent medical treatment of the early stage of syphilis are delayed, this leads to serious complications. Therefore, a high sensitivity and specificity syphilis screening examination that can be administered rapidly with decreased labor costs is required. For the syphilis examination in China several years ago, manual kits, such as point‐of‐care testing (POCT) and ELISA kits, were used in addition to the gold‐standard *Treponema pallidum* particle agglutination (TPPA) test. Furthermore, fully automated chemiluminescent immunoassay reagents have been used as routine assays in hospitals.

Wellinghausen and colleagues reported that the concordance rates for positive and negative serum were calculated for TPPA and chemiluminescent immunoassay kits made by LIAISON (DiaSorin) and Architect Syphilis TP (Archi‐TP), and the positive concordance rates were 100% (18/18), 100% (17/17), and 100% (18/18), respectively. Furthermore, the negative concordance rates were 100%, 99.8%, and 99.6%, respectively. Archi‐TP showed less specificity than the TPPA.^2^ On the other hand, it was reported that the LIAISON kit has higher sensitivity and that TPPA has higher specificity among nine serological TP screening assays, including the LIAISON, Archi‐TP, and TPPA.^3^ Similarly, 149 were judged positive by the Archi‐TP. Thirty‐seven out of 149 samples showed different results according to TPPA and Archi‐TP. Eight were judged (21.6%) as positive, 11 (29.7%) as indeterminate, and 18 (48.6%) as negative by other dot‐blot methods. In this article, further analysis by TPPA after Archi‐TP screening examination is recommended.^4^ It may be necessary to clarify the sensitivity and specificity of commercial anti‐TP reagents.

Among the Treponema pallidum polypeptides, at least five (TpN15, TpN17, TpN37, TmpA, and TpN47) have proved to be of diagnostic relevance.^5^, ^6^ The LIAISON *Treponema pallidum*‐specific and Immulite syphilis screens, however, use only TpN17, suggesting that TpN17 could be the main TP antigen.^7^

Furthermore, the components that are used by the HISCL anti‐TP, Elecsys Syphilis, Archi‐TP, and Chemclin CLIA reagents are TpN15, TpN17, and TpN47.^7^ Therefore, the performance differences of these TP kits might be due to differences in the amount of antigen used.

Lefevre and colleagues showed that for the detection of anti‐TP antibody for each stage of syphilis, anti‐TP IgM was positive for 16/17 cases of primary syphilis, 11/13 of secondary syphilis, 9/14 of early latent syphilis, and 0/33 of late latent syphilis. On the other hand, anti‐TP IgG was positive for 14/17, 13/13, 14/14, and 33/33 of samples at each stage.^8^

Western blotting of IgM against *Treponema pallidum* antigen was performed for 39 pairs of maternal/infant serum. Fetal IgM antibodies in each case were detected specifically. The combined data suggested that fetal serum IgM reactivity with the 47‐KDa antigen of TP could be used as an important molecular marker for the diagnosis of congenital syphilis. It was found that the anti‐TP IgM antibody was positive in the early stage of infection.^9^, ^10^

This study aims to evaluate the diagnostic performance of a new anti‐TP screening kit, Lumipulse G TP‐N assay (Lumi‐TP), comparing with the Archi‐TP and to confirm the judgment by the TP gold standard test, TPPA. Furthermore, the secondary purpose is to investigate the reactivity of the Lumi‐TP and Archi‐TP assays against the TP major antigens TpN15, TpN17, and TpN47 by using an immune‐chromatography kit, ESPLINE TP, to detect two lines for Tp15‐17 and TpN47.

## 2 MATERIALS AND METHODS

### 2.1 Clinical specimens

Clinical serum samples, including 1041 negative samples and 223 positive samples, were collected from July 2016 to February 2017 at Sun Yat‐Sen University. All the positive samples and negatives samples were confirmed by clinical diagnosis. These specimens were obtained from 451 males (35.7%) and 813 females (64.3%). These samples were first examined and classified by Archi‐TP, then by Lumi‐TP and finally confirmed by gold standard assay kit, TPPA. The average age of TP‐positive patients was 49.0 years, and the average age of TP negative patients was 48.1 years.

### 2.2 Materials used for each syphilis testing kit

The principle and the materials used for the syphilis testing kits, including Lumi‐TP (Lumi‐TP, Fujirebio Inc), Archi‐TP (Archi‐TP, Abbott), TPPA (Serodia TP‐PA: TPPA, Fujirebio Inc), ESPLINE TP (Fujirebio Inc), and Western blotting for TP, as shown in below Table 1. The Tp15‐17 antigen is a recombinant protein expressed in E. coli by fusing the N‐terminus of the Tp15 antigen and the C‐terminus of the Tp17 antigen, which are the main antigens of syphilis.

**Table 1:** The principle and the materials used for the syphilis testing kits

	Lumi‐TP	TPPA	ESPLINE TP	Archi‐TP	Western blotting
Solid phase	Tp15‐17 and TpN47 antigens	native TP antigen	Tp15‐17 and TpN47 antigens	recombinant TP antigens (TpN15, TpN17, and TpN47)	Tp15‐17 and TpN47 antigens
Conjugate	ALP‐labeled Tp15‐17 antigen and ALP‐labeled TpN47 antigen		ALP‐labeled Tp15‐17 antigen and ALP‐labeled TpN47 antigen	acridinium‐labeled anti‐human IgG conjugate and acridinium‐labeled anti‐IgM conjugates	Anti‐human IgM coupled with ALP conjugate and anti‐human IgG ALP conjugate
Assay principle	CLIA by two‐step sandwich immunoassay	particle agglutination assay	immunochromatography of sandwich‐type immunoassay	CLIA by two‐step sandwich immunoassay	Two‐step sandwich immunoassay

Note

Tp15‐17 antigen: recombinant protein expressed in E. coli containing the N‐terminus of the Tp15 antigen fused to the C‐terminus of the Tp17 antigen.

TpN47 antigen: recombinant protein expressed in E. coli.

Abbreviation: ALP, alkaline phosphatase.

### 2.3 Lumipulse G TP‐N assay

The Lumipulse G TP‐N (Lumi‐TP) assay is an anti‐TP IgG and IgM qualitative kit used with human serum and plasma that is based on a two‐step sandwich chemiluminescent immunoassay. The Tp15‐17 and TpN47 antigens are bound to ferrite particles (generated in‐house) as a solid phase and coupled with alkaline phosphatase (ALP, Oriental Yeast Corp) as a conjugate. Lumi‐TP assays were performed using the automated immunoassay tool Lumipulse G1200 (Fujirebio Inc). If the assay signal in the specimen was higher than or equal to the cutoff value, the signal was judged as positive.

As Lumi‐TP compares the correlation between the measured value and the distribution of negative specimens with Archi‐TP, the values for Lumi‐TP were recalculated as three digits as was done for Archi‐TP.

### 2.4 Architect Syphilis TP assay

The Architect Syphilis TP (Archi‐TP) assay with microparticles coated with recombinant TP antigens (TpN15, TpN17 and TpN47) and acridinium‐labeled anti‐human IgG and IgM conjugate is a two‐step immunoassay for the qualitative detection of antibody to TP in human serum or plasma using a fully automated immunoassay instrument, Architect (Abbott). The specimen is considered reactive for anti‐TP if the signal is greater than or equal to the cutoff value.

### 2.5 Serodia TP‐PA

The Serodia TP‐PA (TPPA) assay (Fujirebio Inc) uses sensitized colored gelatin particles as carriers of the native TP antigen, which was purified from cultured Nichols strain. After each specimen was added, the gelatin particles bound with native TP antigen, and the degree of purified protein agglutination was observed. This quantitative analysis was carried out by measuring the titer required to change the agglutination degree by using an initial titration scheme for each serum. TPPA was used as a gold standard to measure the performance of all other assays.

### 2.6 ESPLINE TP

**Figure 1:**
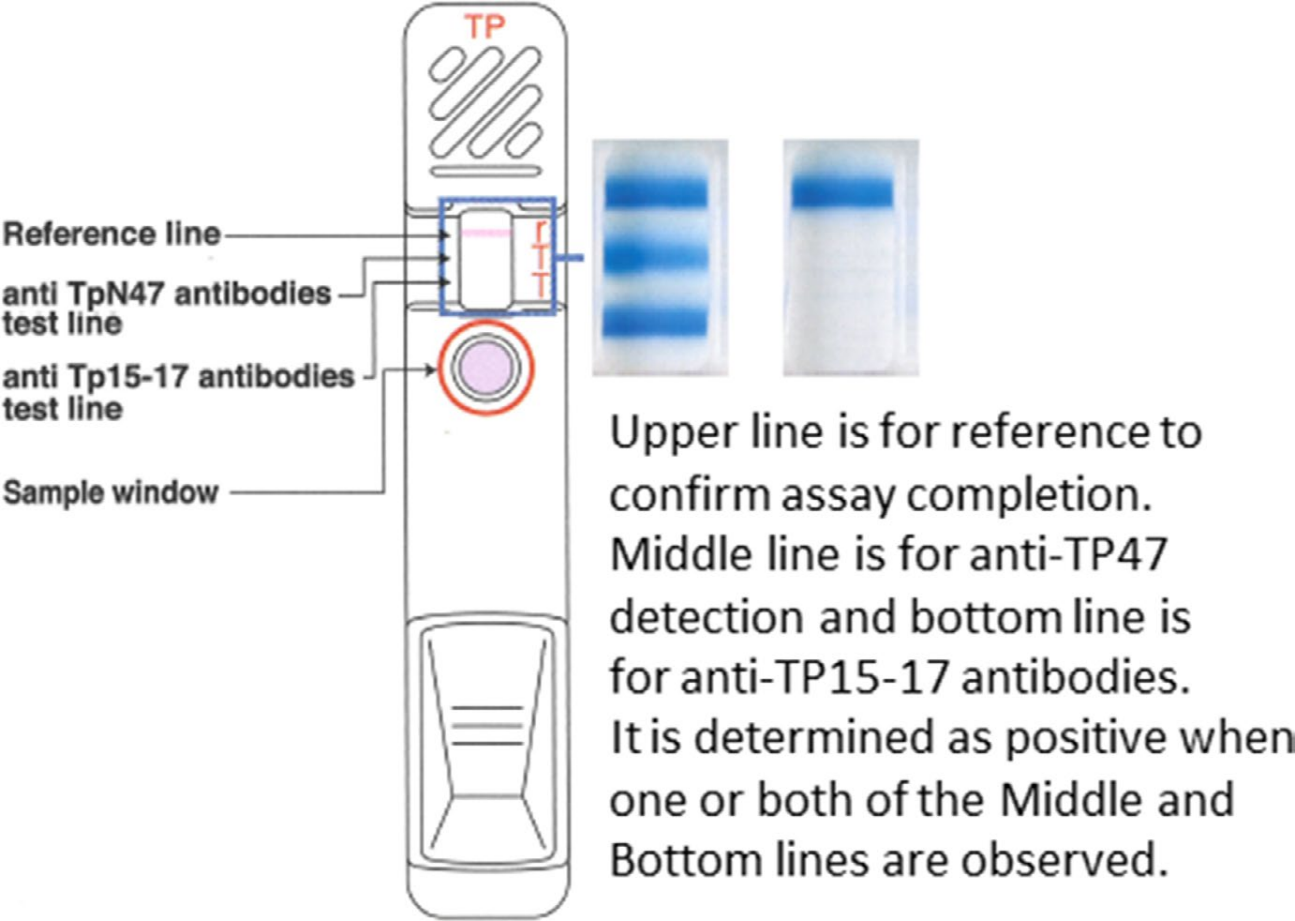
ESPLINE assay principle and result interpretation

ESPLINE TP (Fujirebio Inc) is a syphilis screening test that uses a ready‐to‐use cassette. The chromatography assay is based on a unique sandwich enzyme immunoassay for the detection of IgM and IgG class antibodies against *Treponema pallidum.* The ESPLINE TP assay kit is composed of two lines blotted with the respective Tp15‐17 and TpN47 antigens and a reference line to confirm serum testing on a reaction membrane as a solid phase. Alkaline phosphatase is coupled with the Tp15‐17 and TpN47 conjugates, which are soaked into the pad and dried, and a chromogenic enzyme solution with an alkaline pH is used as an enzyme substrate and development and washing solution. The assay format and procedure are shown in Figure 1. One drop (equivalent to 25 μL) of the sample is dropped onto the specimen area, and the button on the lower portion of the container is pushed, which initiates the assay. TP antibodies in the serum sample form an immunocomplex with the dried ALP‐labeled Tp15‐17 and TpN47 antigens in the specimen area and flow toward the lines on the membrane. These immunocomplexes then reach the lines where the Tp15‐17 and TpN47 antigens are blotted and are captured by them. Thereafter, the chromogenic substrate reaches the lines, and each of the ALP‐labeled immunocomplexes develops color. First, the functioning of the assay is determined by the presence of the colored reference line. The intensity of color development for the two anti‐TP antibody detection lines can be determined as (−), (1+), (2+), or (3+) 15 minutes after the start of the assay.

### 2.7 Western blotting

Western blotting is a manual test used to compare the reactivity of anti‐TP IgG and IgM using membrane‐spotted Tp15‐17 or TpN47. The membrane was blotted with Tp15‐17 and TpN47 antigens (Tp15‐17:0.35 µg/mL, TpN47: 0.42 µg/mL). Serum samples were diluted and added to the membrane. Then, ALP coupled with anti‐human IgG antibodies or anti‐human IgM antibodies (Fujirebio Inc) was used as a secondary antibody. The results of Western blotting are described as (−), (1+), (2+), or (3+). Additionally, (±) was set as the decision threshold.

### 2.8 Performance evaluation study

**Figure 2:**
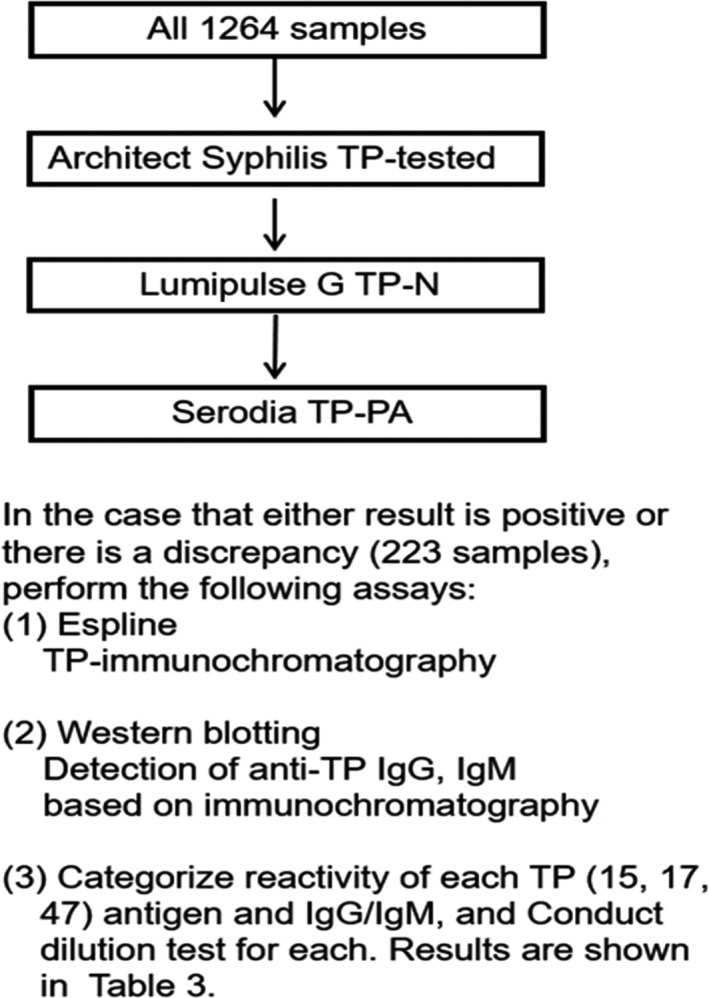
The flowchart of the tests used for the anti‐TP assay kits

Each measured value of Lumi‐TP and Archi‐TP for the 1041 negative specimens was compared according to the frequency distribution for each value range to determine the average value and standard deviation. The coincidence rate of Lumi‐TP, Archi‐TP, and TPPA for 1041 negative and 223 positive specimens was determined by the quadrant table method according to the testing algorithm, as shown in Figure 2. Then, the correlation of the Lumi‐TP and Archi‐TP values for the 223 positive specimens was determined.

### 2.9 Reactivity for Tp15‐17 and TpN47

The Lumi‐TP and Archi‐TP values at each level from (−) to (3+) according to the ESPLINE Tp15‐17 and TpN47 lines were compared. Then, 223 positive specimens with ESPLINE data were classified into four groups as shown below:

Group 1 contained specimens with stronger colored lines for Tp15‐17 than for TpN47.

Group 2 contained specimens with stronger colored lines for TpN47 than for Tp15‐17.

Group 3 contained specimens with weakly colored lines for both Tp15‐17 and TpN47.

Group 4 contained specimens showing discrepancies between the Archi‐TP and Lumi‐TP values.

Group 5 contained other 208 positive specimens.

## 3 RESULTS

When compared to the gold standard reagent TPPA, Lumi‐TP had a sensitivity of 100% (223/223), with a 95% CI of 98.36%‐100.00%, and a specificity of 100% (1041/1041), with a 95% CI of 99.65%‐100%. Archi‐TP showed a sensitivity of 100% (223/223), with a 95% CI of 98.36%‐100%, and a specificity of 99.1% (1032/1041), with a 95% CI of 98.37%‐99.60%. The overall rate of the agreement between Lumi‐TP and TPPA and Archi‐TP and TPPA were 99.7% (*P* < .001) and 97.6% (*P* < .001), respectively, according to Cohen's kappa analysis, as shown in Table 2. The number of false‐positive samples identified by Lumi‐TP and Archi‐TP was 0 (0%) and 9 (0.86%), respectively, and the false‐positive values for Archi‐TP ranged from 1.14 to 2.23.

**Table 2:** Concordance of Lumi‐TP and Archi‐TP Results with TPPA Results

	TPPA		Total
	(+)	(−)	
Lumi‐TP vs TPPA Gold Standard			
Lumi‐TP			
(+)	223	0	223
(−)	0	1041	1041
Total	223	1041	1264
Statistic	Value	95% CI	
Sensitivity	100%	98.36%‐100.00%	
Specificity	100%	99.65%‐100.00%	
Archi‐TP vs TPPA Gold Standard			
Archi‐TP			
(+)	223	9	232
(−)	0	1032	1032
Total	223	1041	1264
Statistic	Value	95% CI	
Sensitivity	100%	98.36%‐100.00%	
Specificity	99.14%	98.37%‐99.60%	

**Figure 3:**
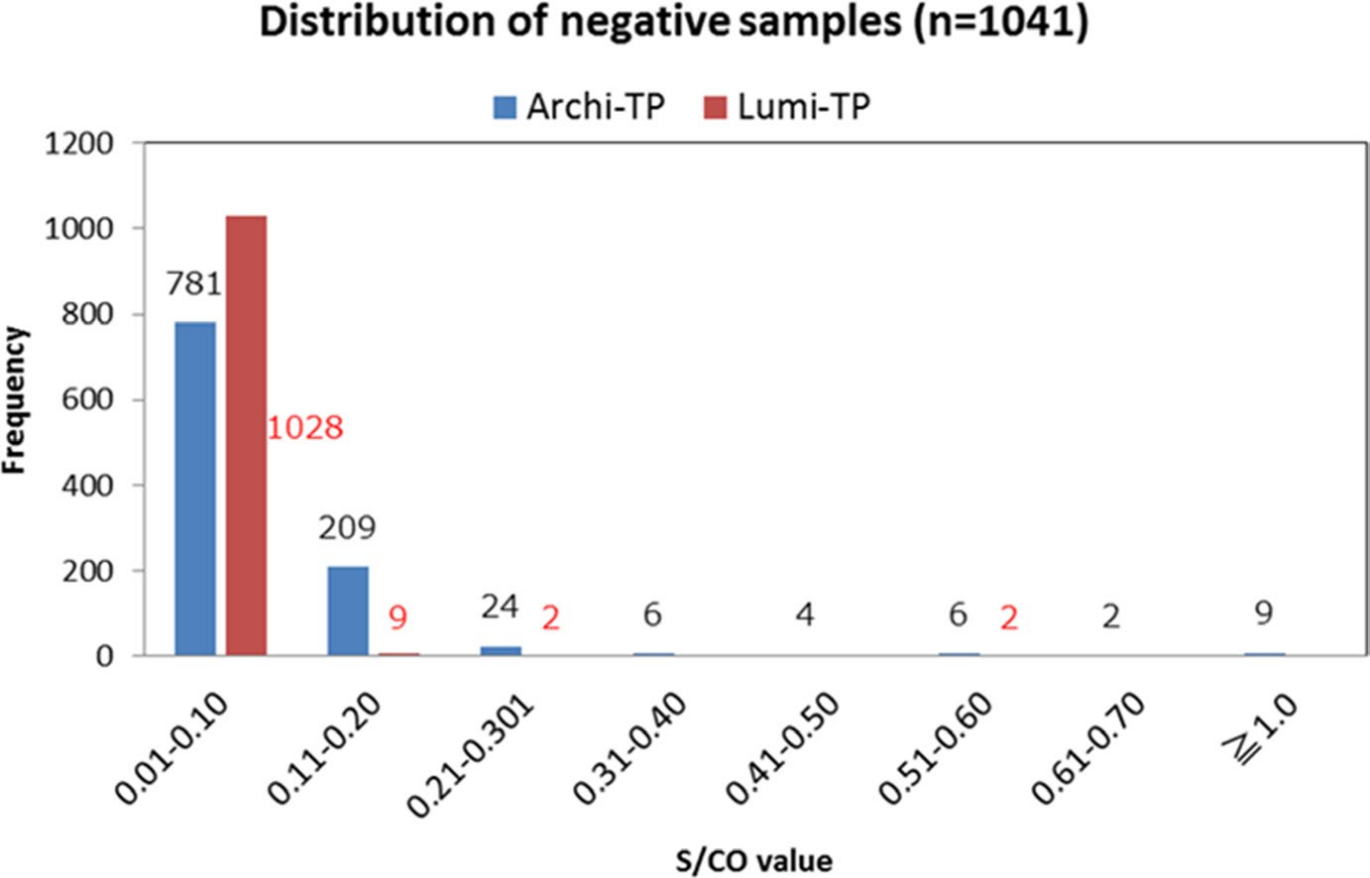
Distribution of negative samples according to Archi‐TP and Lumi‐TP

Figure 3 shows the distribution of the negative sample values for Lumi‐TP and Archi‐TP. The mean values for Lumi‐TP and Archi‐TP were 0.102 (SD 0.025) and 0.102 (SD 0.148), respectively (Figure 3). These results show that the values measured by Lumi‐TP were statistically less dispersed than those measured by Archi‐TP. The mean value plus 5 SDs and 10 SDs in terms of the reference cutoff was 0.229 and 0.355, respectively, for Lumi‐TP and 0.845 and 1.587, respectively, for Archi‐TP.

**Figure 4:**
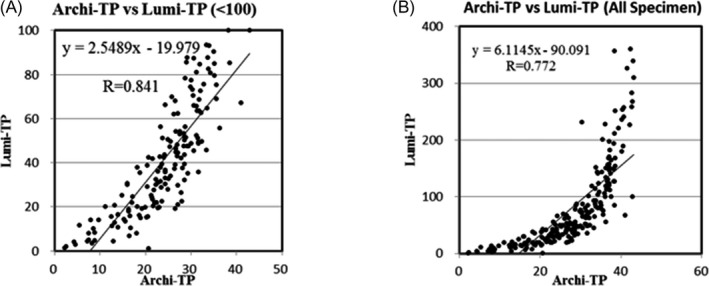
Correlation of Archi‐TP and Lumi‐TP results for each value range. A, For value range of 100 or less by Lumi‐TP, (B) for all specimens

Correlation tendency and rate between Archi‐TP and Lumi‐TP were 2.549 and 0.841 for 160 positive specimens (Figure 4A), and Archi‐TP reached a plateau at about 40 of value for specimens with higher levels than 160 in Lumi‐TP. Furthermore, these values were 6.115 and 0.772 for all specimens (Figure 4B).

The Lumi‐TP and Archi‐TP values were divided into groups according to the band intensity of the ESPLINE Tp15‐17 and TpN47 lines. Eleven specimens lines for Tp15‐17 stronger colored than for TpN47 (group 1). For sample P170, Archi‐TP showed a slightly higher value than Lumi‐TP, and Western blotting of Tp15‐17 IgM showed strong reactivity. On the other hand, for samples P172 and P188, Lumi‐TP showed higher values than Archi‐TP, and Western blotting of Tp15‐17 IgG showed strong reactivity. Lumi‐TP showed a higher value for P44. For this specimen, a slightly more strongly colored line for TpN47 and positive Western blotting results for TpN47 IgM were obtained (group 2). Then, two specimens with weakly colored lines for both Tp15‐17 and TpN47 (group 3) showed almost the same values. Among the 223 positive specimens, one specimen with a discrepancy showed a 1.00 COI for Lumi‐TP, a 20.64 COI for Archi‐TP, a 1+ value for the Tp15‐17 band for ESPLINE TP, and a 2+ value according to the anti‐TP Western blotting results, and it was also shown to be positive at up to a 640‐fold dilution according to TPPA (Table 3).

**Table 3:** Each measurement value for a typical specimen using each reagent

	Archi‐TP	Lumi‐TP	TPPA	ESPLINE		Western blotting			
				TpN47	Tp15‐17	TpN47		Tp15‐17	
						IgM	IgG	IgM	IgG
Group 1									
P2	9.71	14	640	1+	2+	−	−	+	+
P26	24.28	30.5	640	1+	2+	−	−	+	2+
P41	2.41	2.1	320	−	2+	−	−	+	−
P109	8.69	4.9	320	1+	2+	−	−	−	−
P59	7.17	4.6	320	1+	2+	−	−	−	+
P63	7.49	5.2	320	1+	2+	−	−	+	+
P170	8.18	3.4	320	−	2+	−	−	2+	+
P172	16.01	30.6	640	1+	2+	−	−	−	2+
P178	18.84	14.9	320	1+	2+	+	−	+	+
P188	24.11	43.8	640	1+	2+	−	−	+	2+
P209	12.88	4.5	640	−	2+	−	−	+	+
Group 2									
P44	5.39	11.8	320	2+	1+	+	−	−	−
Group 3									
P106	2.2	1.4	160	1+	1+	−	−	+	−
P164	4.47	3	320	1+	1+	−	−	+	+
Group 4									
P205	20.64	1	640	−	1+	+	2+	+	2+

Note

The 223 positive cases were divided into five groups by comparing the color intensity of the two positive ESPLINE bands.

Group 1 contained specimens with stronger colored lines for Tp15‐17 than for TpN47.

Group 2 contained specimens with stronger colored lines for TpN47 than for Tp15‐17.

Group 3 contained specimens with weakly colored lines for both Tp15‐17 and TpN47.

Group 4 contained specimens showing discrepancies between the Arch‐TP and Lumi‐TP values.

## 4 DISCUSSION

Lumi‐TP appears to show better diagnostic performance in terms of specificity than Archi‐TP, as shown by the narrow distribution of the values for the negative samples and the correlation of the TP testing results with those of the gold standard assay, TPPA. Lumi‐TP has a higher margin for the cutoff value as a result. Therefore, for Lumi‐TP, the certainty of the test results for anti‐TP positive samples may be increased. Then, Lumi‐TP might lead to a reduced need for retesting.

There was a report that the use of anti‐human antibodies tended to cause the deterioration of specificity and an increase in false positives due to the influence of nonspecific components, such as rheumatoid factors.^11^ The use of anti‐human antibodies in reagents for the detection of *Treponema pallidum* in syphilis might result in the degradation of specificity.

In terms of the accuracy of the measurement of low concentrations of anti‐TP antibodies, although anti‐TP antibody values are not utilized for monitoring the medical condition of patients, Lumi‐TP values might be able to be used for the qualitative detection of anti‐TP antibodies and the early stage of syphilis. Based on the correlation of the Lumi‐TP and Archi‐TP results, the Archi‐TP values reached a plateau at a concentration of 40 or more according to the Lumi‐TP results, as shown in Figure 4B. Since Lumi‐TP can measure anti‐TP antibodies within a wide range, it is suggested to investigate the relationships between anti‐TP antibody levels, syphilis stage, and determination.

In the eight specimens in group 1 of Table 3 tested with ESPLINE that showed Tp15‐17(+) and TpN47 (+) bands and stronger Tp15‐17 staining, four cases showed higher Archi‐TP values, and four cases showed higher Lumi‐TP values. In three cases with only Tp15‐17 staining, Archi‐TP showed a higher value than Lumi‐TP. Therefore, it is considered that although the reactivity of Lumi‐TP and Archi‐TP against anti‐TP antibodies are similar, Archi‐TP mainly detects antibodies against TpN15 and TpN17, and Lumi‐TP mainly detects antibodies against Tp15‐17 and TpN47.

On the other hand, in one specimen in group 2 shown in Table 3 that showed stronger ESPLINE staining for the TpN47 band, Lumi‐TP showed higher values. This also indicates that Lumi‐TP is highly reactive for the TpN47 antigen. In the early stage in syphilis, there were more specimens with detection of anti‐TP IgM than those with detection of anti‐TP IgG.^9^ On the other hand, it has been reported that TpN47 is highly reactive in the early stage of syphilis or in patients with a previous infection.^6^, ^12^ It is possible that Lumi‐TP might detect anti‐TP antibodies in the infectious early stage.

According to Western blotting, eleven cases in groups 1‐4, as shown in Table 3, were positive for Tp15‐17‐IgM, one of which showed intense staining. As Western blotting showed stronger anti‐IgG staining for Tp15‐17 and weaker anti‐IgM staining for Tp15‐17, these cases might be only in the early stage of syphilis infection^13^

Regarding the reactivity of specimens from patients in the early stage of syphilis infection, it is necessary to carry out further comparative examinations using the various assay reagents on specimens having IgM type TP antibodies in cooperation with hospitals or diagnostic facilities treating patients in the early infectious stage.

The results for the P205 sample showed a discrepancy in that they showed a high Archi‐TP value of 20.67 and a low Lumi‐TP value of 1.0. The values obtained from the TPPA and ESPLINE assays for this specimen were 640 and a weak positive line (+1) for ESPLINE Tp15‐17, respectively. When arranged in order of assay intensity, the order is Arch‐TP, TPPA, ESPLINE TP, and Lumi‐TP. The Archi‐TP detection method uses anti‐human IgG and anti‐human IgM antibodies, while other methods use a double‐antigen method that uses TP antigen as the solid phase and a labeled conjugate. The discrepancies might be due to differences in the assay formats, although further study will be required to confirm this.
